# Hydrothermally Treated Chitosan Hydrogel Loaded with Copper and Zinc Particles as a Potential Micronutrient-Based Antimicrobial Feed Additive

**DOI:** 10.3389/fvets.2015.00062

**Published:** 2015-11-23

**Authors:** Parthiban Rajasekaran, Swadeshmukul Santra

**Affiliations:** ^1^NanoScience Technology Center, University of Central Florida, Orlando, FL, USA; ^2^Department of Chemistry, University of Central Florida, Orlando, FL, USA; ^3^Department of Materials Science and Engineering, University of Central Florida, Orlando, FL, USA; ^4^Burnett School of Biomedical Sciences, University of Central Florida, Orlando, FL, USA

**Keywords:** chitosan, copper, zinc, antimicrobial feed additive, antimicrobial resistance, micronutrient

## Abstract

Large-scale use of antibiotics in food animal farms as growth promoters is considered as one of the driving factors behind increasing incidence of microbial resistance. Several alternatives are under investigation to reduce the amount of total antibiotics used in order to avoid any potential transmission of drug resistant microbes to humans through food chain. Copper sulfate and zinc oxide salts are used as feed supplement as they exhibit antimicrobial properties in addition to being micronutrients. However, higher dosage of copper and zinc (often needed for growth promoting effect) to animals is not advisable because of potential environmental toxicity arising from excreta. Innovative strategies are needed to utilize the complete potential of trace minerals as growth promoting feed supplements. To this end, we describe here the development and preliminary characterization of hydrothermally treated chitosan as a delivery vehicle for copper and zinc nanoparticles that could act as a micronutrient-based antimicrobial feed supplement. Material characterization studies showed that hydrothermal treatment makes a chitosan hydrogel that rearranged to capture the copper and zinc metal particles. Systemic antimicrobial assays showed that this chitosan biopolymer matrix embedded with copper (57.6 *μ*g/ml) and zinc (800 *μ*g/ml) reduced the load of model gut bacteria (target organisms of growth promoting antibiotics), such as *Escherichia coli*, *Enterococcus faecalis*, *Staphylococcus aureus*, and *Lactobacillus fermentum* under *in vitro* conditions. Particularly, the chitosan/copper/zinc hydrogel exhibited significantly higher antimicrobial effect against *L. fermentum*, one of the primary targets of antibiotic growth promoters. Additionally, the chitosan matrix ameliorated the cytotoxicity levels of metal supplements when screened against a murine macrophage cell line RAW 264.7 and in TE-71, a murine thymic epithelial cell line. In this proof-of-concept study, we show that by using chitosan as a delivery platform, micronutrient-based metal feed additives could be used to minimize the undesirable levels of microbial population without causing significant cytotoxic effect under *in vitro* conditions. These findings provide the platform for further studies in target animal models to quantify the required physiological concentrations of copper and zinc when delivered via a chitosan hydrogel platform to elicit a growth promoting effect without causing any toxicity.

## Introduction

The World Health Organization recently cautioned that antimicrobial resistance amongst infectious agents has reached alarming levels and that it could turn the twenty-first century into a time where common wound injuries can become lethal infections (1). Over use and unregulated use of antibiotics in the past six decades has created a selection pressure for drug resistant microbes (2, 3). Particularly, food animal production facilities, such as cattle, swine, chicken, and fish farms, use subtherapeutic levels of antibiotics as antimicrobial growth promoters (AGPs) in tons scale in developing countries and some developed countries including United States (3, 4). AGPs are used for various benefits including high feed conversion ratio, control of zoonotic infections, and for prevention of herd infections among closely housed animals in intensive farm animal production facilities (5, 6). Various alternatives, such as antimicrobial peptides, recombinant enzymes, plant extracts, and heavy metals, such as copper and zinc salts, are used as growth promoting feed supplements to reduce the usage on classical antibiotics (7).

Copper and zinc are essential micronutrient minerals that play important roles in physiological processes at lower concentrations and so the National Research Council (NRC) recommends them as minor feed supplements in cattle and swine production (8). However, when used at elevated levels as feed additive, copper in the form of copper sulfate (CuSO_4_) and zinc in the form of zinc oxide (ZnO) have shown to produce a growth promoting effect via reduction of fermentation loss of energy by altering the gut microbiome (9). Higher feeding levels of copper and zinc also have shown to suppress gut pathogens, thereby, posing themselves as a potential substitute for traditional antibiotics in industrial food animal production facilities (8). Despite these advantages, extended and frequent administration of such high doses of copper and zinc salts (Table 1) would predispose gut microbes to develop resistance not only against these metals but also to traditional antibiotics that share similar microbe killing mechanisms (10). Further, higher levels of copper and zinc in the animal excretory wastes may pose serious threat to the soil microbial diversity and the surrounding ecosystem. Thus, developing a strategy that can considerably reduce the frequency of administration of copper and zinc in feed, yet maintaining the growth beneficial effect would be a viable alternative. One such strategy would be to increase the gastric retention time of the supplied minerals by embedding the metallic salts in a biocompatible polymer that has affinity for the gastro intestinal tract.

**Table 1:** **Current levels of copper and zinc usage as feed additives in food animal farms (references for the values in parenthesis next to the numbers)**.

Daily feed (kg/day)	Copper (mg/kg diet)	Zinc (mg/kg diet)
Cattle (5.5–10)	10 (8)	30 (8)
Swine (1–2)	100–250 (11)	2000–3000 (11)
Poultry (0.1–0.2)	4–8 (12)	30–60 (12)

Chitosan is one such biopolymer that consists of long chains of N-acetyl-d-glucosamine molecules and has been investigated extensively for its potential as an animal feed additive in poultry, swine (13, 14) and also in cattle (15, 16). Chitosan and its oligosaccharide derivatives have been shown to promote body weight gain of poultry and swine by enhancing nutrient digestibility, increasing villus height and villus/crypt ratio in the illea and jejuna (13, 14). Additionally, chitosan microparticles have been shown to reduce the shedding of pathogenic *Escherichia coli* strain O157:H7 from the intestinal tract of cattle when supplemented with feed (15). Though, the differences in effect if any, on the gastrointestinal health is not currently available, nevertheless, chitosan appears to be a viable feed additive for ruminants (16) as much as it for monogastric animals (13). Copper-loaded chitosan nanoparticles have been shown to possess beneficial effect on the intestinal health of weaned piglets (14). Similarly, a chitosan–zinc chelate was shown to improve intestinal structure in weaned piglets (17). In all these studies, chitosan was used as either a chelating agent or as an encapsulating micro or nanoparticle for either copper or zinc salt individually. All these strategies would not provide a significant advantage in terms of gastric retention time over the current practice of feeding a salt mixture. Also, uniform loading of both metals in a chelate or inside an encapsulated particle is challenging.

We recently demonstrated that hydrothermal (HT) treatment of chitosan results in depolymerization of chitosan into shorter chain polymers with increased functional groups availability for loading high amounts of antimicrobial copper and similar metals (18). Furthermore, highly depolymerized chitosan possesses exceptional muco-adhesive property owing to the presence of high net positive charge provided by the amine group in the linear polysaccharide matrix (13). We also showed that the increased functional groups after HT treatment help in enhancing the dispersibility of chitosan in water, thus significantly potentiating the usefulness as a feed additive by overcoming the bottleneck of insolubility (18). By taking advantage of the above-mentioned merits, herein, we developed a chitosan hydrogel matrix that is loaded with both copper and zinc metal nanoparticles that can be used as a potential micronutrient-based antimicrobial feed additive. In this proof-of-concept manuscript, we demonstrate the antimicrobial ability of this chitosan–metal composite by screening against model organisms for gut bacteria that are targets of conventional antibiotic growth promoters. We also show how loading of copper and zinc together in a chitosan hydrogel matrix minimized the cytotoxic effects of the metals on eukaryotic cells (model for host tissues). We infer that this report will be the foundation for our future studies that will examine this novel material for its growth promoting effects involving target animals.

## Materials and Methods

### Synthesis of Chitosan–Metal Composites

One pot HT method of making water-dispersible chitosan–metal composite hydrogel was performed as described earlier (18). Briefly, low molecular weight chitosan (Sigma-Aldrich, St. Louis, MO, USA) was dissolved in 1% HCl at 10 mg/ml and HT depolymerization was done inside a preheated Lindeberg/Blue M oven (Thermoelectron Corporation) at 150°C for 90 min. Copper sulfate (10 mg/ml) (CQ concepts Inc, Ringwood, IL, USA) and zinc nitrate (100 mg/ml) were added to the chitosan right after HT treatment. After overnight stirring, the pH was raised to 7.4 (by adding 1 N NaOH solution) before material characterization, cytotoxic, and antimicrobial studies. The abbreviated code names of the various samples that were used in this experiment and their corresponding contents are provided in Table 2.

**Table 2:** **Various samples and their abbreviations that were used in this experiment**.

Sample name	Contents
HTChitosan	Hydrothermal treated chitosan (in 1% HCl)
HTCh-RTCuZn	Copper sulfate and zinc nitrate at room temperature mixed with HT chitosan
RTCuZn	Mixture of copper sulfate and zinc nitrate in 1% HCl with no HT treatment (no chitosan)
HTCh-RTCu	Copper sulfate mixed with HT chitosan
HTCh-RTZn	Zinc nitrate mixed with HT chitosan

### Scanning Electron Microscopy

A Zeiss ULTRA-55 FEG SEM was used to study the gross morphological characteristics of the chitosan–metal complex. The liquid samples were spin coated (2000 rpm) on boron-doped silicon wafers (Nova electronic materials) and the images were acquired at 5 kV.

### High-Resolution Transmission Electron Microscopy

Carbon-filmed gold grids (400 square mesh) (Catalog # CF-400-Au, Electron Microscopy Sciences, Hatfield, PA, USA) were used for preparing samples where the materials were dip coated and air dried at room temperature before they were analyzed in a FEI Tecnai F30 microscope at the Material Characterization Facility (MCF) at the University of Central Florida. An electron beam intensity of 100 kV was used for studying all the materials.

### Bacterial Growth Media and Conditions

The growth inhibition effect of the chitosan–metal composites was tested against a range of microorganisms that acted as model organisms for gut microflora of livestock gastrointestinal tract. *E. coli* (ATCC 8739) and *Staphylococcus aureus* (ATCC 25923) were grown in tryptic soy media (Sigma-Aldrich). *Enterococcus faecalis* (ATCC 29212) was grown in brain heart infusion (BHI) media (BD Biosciences). *Lactobacillus fermentum* (ATCC 9338) was grown in MRS medium (BD Biosciences). All incubations were done at 37°C.

### Antimicrobial Assays

Initial screening for determining the most effective metallic concentration was performed using a microplate alamar blue assay (MABA) as described elsewhere (18). The MABA assay was used as a substitute for broth microdilution assay to determine minimal inhibitory concentration (MIC) to circumvent the interference of the chitosan hydrogel in light absorbance. Briefly, the chitosan–metal hydrogel were added in triplicates for each concentration considered in a flat bottom 96-well plate. The volume of the sample hydrogel was kept at 20 *μ*l in the final volume of 200 *μ*l in each well. As per clinical and laboratory standards institute (CLSI) requirements, for all the bacterial species, a culture concentration of 0.5 McFarland standards was used as a starter culture and from which 1 × 10^5^ CFU/well (final concentration 5 × 10^5^ CFU/ml) was added. A classical antibiotic kanamycin (50 *μ*g/ml) was used as positive control for killing. After 24 h of incubation at 37°C, 10 *μ*l of alamar blue dye (Molecular probes, Eugene, OR, USA) was added to each well. The plate was then kept back in the incubator for one more hour before the absorbance was measured at both 570 and 600 nm for each well. The reduction of the dye in percentage (correlates to metabolically viable bacteria) value was calculated by using the formula as suggested by the manufacturer. The motivation behind choosing these specific concentrations of metals (copper and zinc) in these assays was to ascertain the difference in antimicrobial activity against gut microbes between the traditional method (no chitosan) and using chitosan hydrogel matrix as a delivery system.

### Bacterial Killing Assay

The absolute values of the reduction in bacterial numbers after treatment with various concentrations of chitosan–metal composite were determined using bacterial killing assay (CFU assay). The protocol used in the MABA assay was followed for this assay for treatment of bacteria with samples but at the end of 24 h incubation, serial dilutions of the bacteria from each well were made in phosphate-buffered saline and plated on corresponding agar plates. The colonies were counted after overnight incubation and expressed in logarithmic scale.

### Cytotoxicity Experiments

To determine any potential cytotoxicity effects of the newly synthesized chitosan–metal hydrogel, RAW 264.7 murine macrophage and TE-71 mouse thymic epithelial cell lines were treated with various concentrations (6.25, 12.5, 25, and 50 *μ*g/ml of metallic zinc concentration) of the hydrogel. These numbers were selected to identify the specific concentration where there is a difference in cytotoxicity levels between the samples that contained chitosan and those that did not. (The RAW 264.7 macrophages were a kind gift from Dr. J. Manuel Perez at University of Central Florida.) After 24 h of incubation at 37°C in the presence of 5% CO_2_, the media containing hydrogel were removed and fresh media [DMEM (Corning, 10-090-CV) complete media containing 10% fetal calf serum and 1% antibiotic and antimycotic] were added to avoid any interference from the material during measurement of fluorescence. Then, to each well in the 96-well flat bottom plates, 20 *μ*l of alamar blue reagent was added. After 3 h of incubation with the alamar blue reagent, the contents of the plates were transferred to black 96-well plate (Costar 3916, Corning life Sciences) and were read for fluorescence (590 nm) in a TECAN infinite M200PRO plate reader. Triplicate wells were used for each sample and the wells containing only the macrophages and media (no samples added) were used as growth control. The percentage viability of cells in the chitosan–metal samples treated wells were deduced by normalizing the viable cells percentage in the growth control wells.

### Statistical Analysis

To determine the statistical significance of the difference between treated samples and their corresponding controls in antimicrobial and cytotoxicity experiments, ANOVA and Tukey’s honestly significant difference methods were used. For all experiments, *P* < 0.05 was considered significant.

## Results

### HT-Treated Chitosan Hydrogel Rearranges to Capture Copper and Zinc Nanoparticles

**Figure 1:**
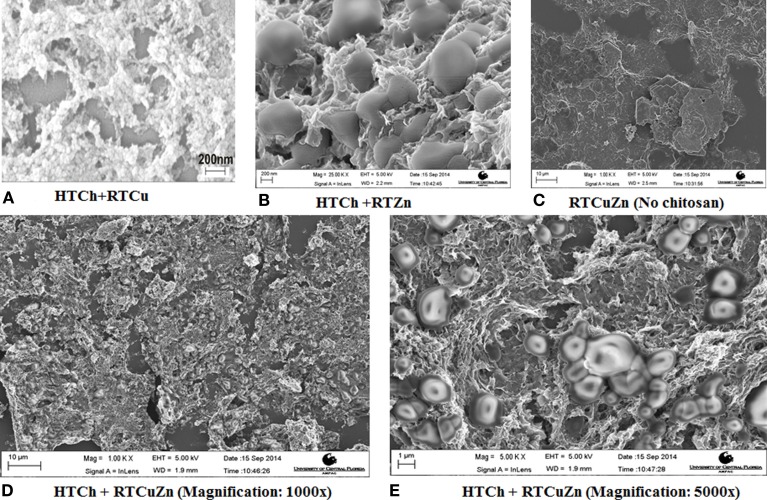
**SEM images of chitosan–metal hydrogel**. SEM images showing metal particles (round shaped) embedded uniformly on to a chitosan matrix background **(A,B,D,E)** when compared to the sample that have no chitosan **(C)** which appeared to be a mixture of salt solution with no specific morphological features. The chitosan appears in **(A–E)** as a mesh (3-D structure) where the metal particles (rounded structures of ~1–2 *μ*m in diameter) are embedded in between the hydrogel matrix.

The scanning electron microscopy (SEM) image of the chitosan–metal hydrogel showed that the copper and zinc nanoparticles (of size <5 nm) were uniformly distributed throughout the HT chitosan matrix background (Figures 1D,E). Adding copper (Figure 1A) and zinc (Figure 1B) either individually or together (Figures 1D,E) did not affect the distribution characteristics of the metal nanoparticles. The control samples and the metal salt mixture (Figure 1C) did not appear to possess any specific morphological features (no chitosan) suggesting that it was a salt mixture. However, in the presence of chitosan, the metal particles can be seen as rounded nanostructures embedded in a hydrogel matrix of chitosan in respective samples (1A–1E). Using dynamic light scattering technique, a polydispersity index of 1.0 was observed for the chitosan–metal hydrogel that verified the finding that they were a hydrogel and not individual particles (data not shown).

### Multiple Forms and Oxidation States of Metal Salts were Embedded in the Chitosan Hydrogel

**Figure 2:**
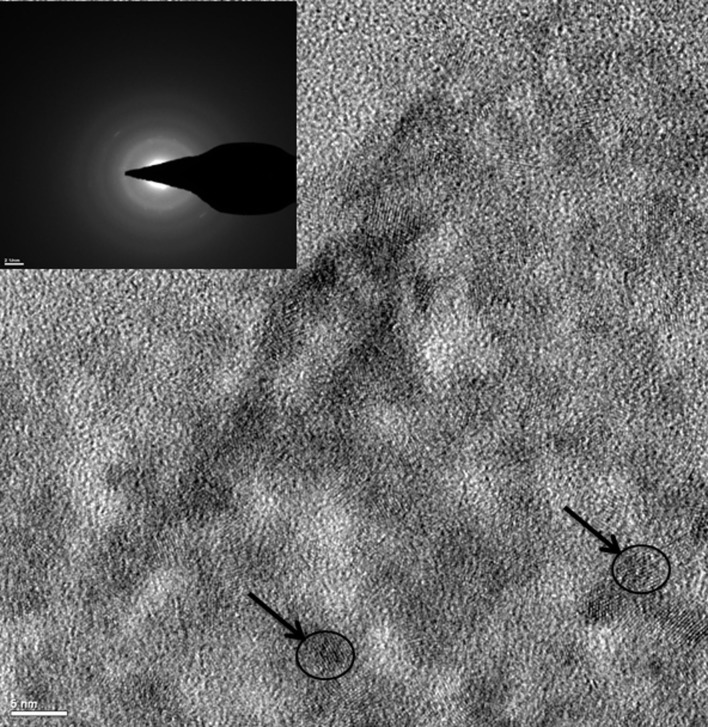
**TEM image of HTCh + RTCuZn**. Under a transmission electron microscope, the rounded metal particles appear as crystalline structures (indicated by arrows and circles) embedded on an amorphous chitosan hydrogel matrix. Inset picture showing the lattice spacing of the metal crystallites (indicated by arrows in the pictures). The “*d*” spacing (identified by measuring the diameter of the white rings in the inset picture) measured using the inset picture suggested that the metal crystallites were in multiple salt forms and oxidation states. The size of the metal crystallite particles was at 5 nm in diameter (indicated by arrows and circles).

Using selective area electron diffraction (SAED) on the high-resolution transmission electron microscopy (HRTEM) images, the lattice spacing of the metal nanocrystallites (Figure 2) were analyzed to determine the form and oxidation states of the metals in the hydrogel. The different forms and oxidation states of the metals were determined by the measuring the “*d*” spacing in the SAED pattern (inset of Figure 2). The metals in the hydrogel composite were present as metallic copper, copper oxides (CuO and Cu_2_O), copper chloride, copper zinc alloy, metallic zinc, zinc oxide, zinc nitrate, and zinc chloride (Figure 2). HRTEM analysis also showed that the size of the metal nanoparticles embedded in the chitosan hydrogel was in the range of 5 nm (indicated by arrows in Figure 2).

### HT Chitosan Significantly Enhances the Antimicrobial Efficacy of Metals Against the Fermenter Group Bacteria *L. fermentum*

**Figure 3:**
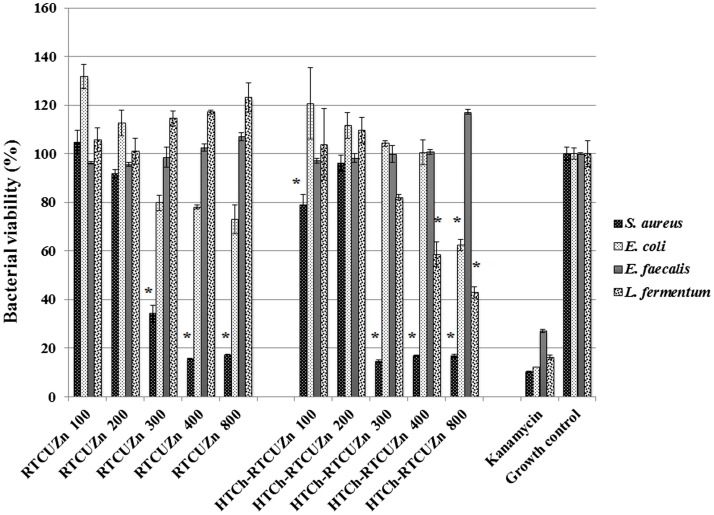
**Microplate alamar blue assay (MABA)**. Sample name explanation: RTCuZn – mixture of copper sulfate and zinc nitrate in 1% HCl with no HT treatment (no chitosan); HTCh-RTCuZ – copper sulfate and zinc nitrate at room temperature mixed with HT chitosan. HTCh-RTCuZn exhibited superior bacterial killing effect at 800 *μ*g/ml of metallic zinc concentration when screened against all the four model gut bacteria. The numbers in the sample labels represent metallic zinc concentration at 100, 200, 300, 400, and 800 *μ*g/ml. The corresponding chitosan and copper concentrations in those samples are 7.2, 14.4, 21.6, 28.8, and 57.6 *μ*g/ml, respectively. Kanamycin (50 *μ*g/ml) was used as control for bacterial killing. MABA was used as a substitute for broth microdilution assay to screen for effective concentration. The absorbance values of all the test samples were normalized for control samples, i.e., the percentage of viable cells in the growth control is considered as the actual bacterial numbers after 24 h of incubation when there were no antimicrobials added. Higher percentage (than control samples) noticed in some samples corresponds to higher metabolic activity in those samples and the actual number of bacteria is presented in Figure 4. The alamar blue dye undergoes extinction (at a very slow rate) when exposed to light and ambient temperature in the absence of any reducing agent, such as bacteria, and that is observed (~10–20%) in the kanamycin-labeled columns though there were no viable bacteria in there as expressed in Figure 4. [* indicates significant difference between the treatment and respective growth control groups (*P* < 0.05)].

**Figure 4:**
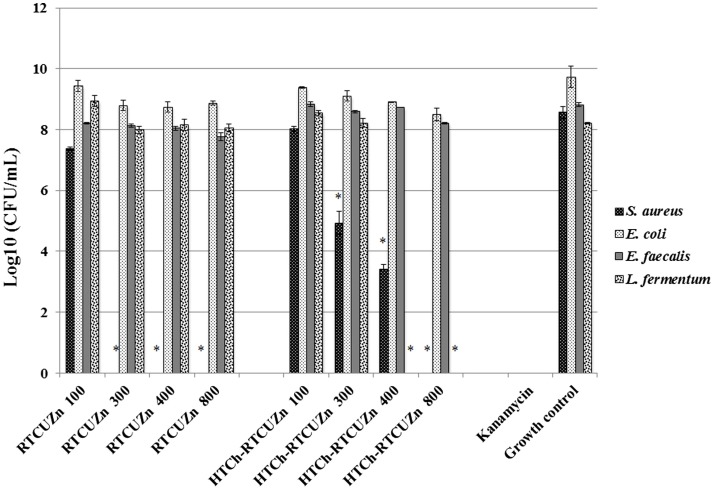
**Colony-forming unit (CFU) assay**. Absolute bacterial numbers in logarithmic scale after treatment with either HTCh-RTCuZn or RTCuZn for 24 h. Sample name explanation: RTCuZn – mixture of copper sulfate and zinc nitrate in 1% HCl with no HT treatment (no chitosan); HTCh-RTCuZ – copper sulfate and zinc nitrate at room temperature mixed with HT chitosan. The numbers in the sample labels represent metallic zinc concentration at 100, 300, 400, and 800 *μ*g/ml. The corresponding chitosan and copper concentrations are 7.2, 21.6, 28.8, and 57.6 *μ*g/ml, respectively. At 800 *μ*g/ml of metallic zinc concentration, the reduction in bacterial load in the samples treated with HTCh-RTCuZn was >99.99% in *S. aureus* and *L. fermentum* compared to their corresponding growth control group. In *E. coli*, the percentage reduction was 94.95% and in *E. faecalis* the reduction was 75.55%. Kanamycin (50 *μ*g/ml) was used as control for killing. [* indicates significant difference between the treatment and respective growth control groups (*P* < 0.05)].

In both MABA and CFU assay, the chitosan hydrogel loaded with copper and zinc salts showed an enhanced killing effect (>99.99%) on *L. fermentum* (Figures 3 and 4) at metallic zinc concentrations of 400 and 800 *μ*g/ml when compared to the salt mixture at same concentrations, where it inhibited bacterial growth by only 42.4 and 56.6%, respectively. The corresponding copper and chitosan concentrations were 28.8 and 57.6 *μ*g/ml. The chitosan hydrogel appeared to be superior in specifically reducing the *L*. *fermentum* of bacteria compared to the salt mixture (no chitosan).

### Chitosan Hydrogel Did Not Affect the Antimicrobial Effect of Metal Salts on the Opportunistic Pathogen Load

For this study, we used *E. coli*, *S. aureus*, and *E. faecalis* as representative organisms of zoonotic and opportunistic pathogens. At 800 *μ*g/ml of metallic zinc (Figures 3 and 4), there was complete inhibition of growth of *S. aureus* (>99.99% reduction of bacteria), while there was a 1.24 log reduction of *E. coli* growth (94.95% reduction of bacteria) by HTCh-RTCuZn. At 800 *μ*g/ml, the metallic zinc containing chitosan–metal hydrogel reduced the load of *E. faecalis* only by 0.61 logs (75.55% reduction), whereas RTCuZn (no chitosan) reduced the load by 90.91%. Also, the salt mixture completely inhibited (>99.99%) growth of *S. aureus* even at 300 *μ*g/ml concentrations. Although not significantly, the growth inhibitory effect of salt mixture was lower in *E. coli* (91.58% reduction) at 800 *μ*g/ml of metallic zinc when compared to HTCh-RTCuZn (94.95%).

### HT Chitosan Matrix Minimized the Cytotoxicity Effects of Antimicrobial Metal Salts

**Figure 5:**
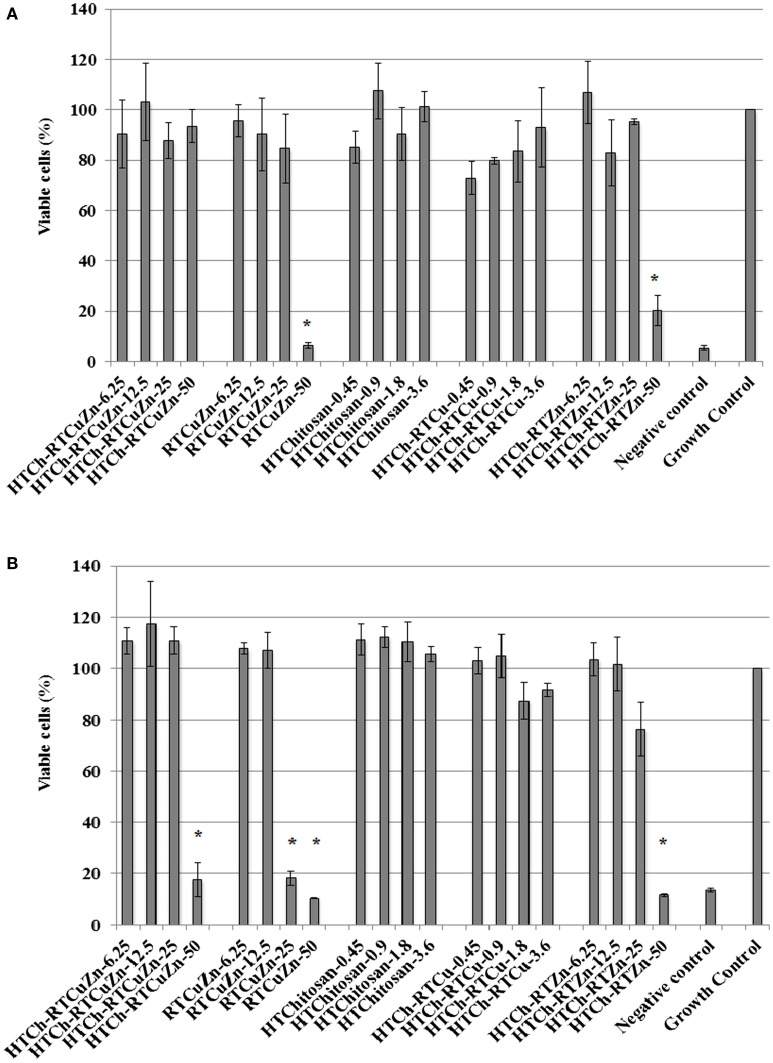
**Cytotoxicity assay: cytotoxicity assay results showing the viability percentage of RAW 264.7 macrophages (A) and TE-71 thymic epithelial cell lines (B) after treatment with various chitosan hydrogel containing metal samples for 24 h**. Sample name explanation: RTCuZn – mixture of copper sulfate and zinc nitrate in 1% HCl with no HT treatment (no chitosan); HTCh-RTCuZ – copper sulfate and zinc nitrate at room temperature mixed with HT chitosan; HTChitosan – hydrothermal treated chitosan (in 1% HCl); HTCh-RTCu – copper sulfate mixed with HT chitosan; HTCh-RTZn – zinc nitrate mixed with HT chitosan. **(A)** In RAW 264.7 macrophage, at 50 *μ*g/ml of metallic zinc concentration, HT chitosan appears to provide protective effect to macrophages, thereby reducing any potential toxicity caused by higher concentration of metallic salts. **(B)** In case of TE-71 epithelial cells, the metals were toxic at 25 *μ*g/ml, while the chitosan ameliorated the toxicity at the same concentration in the HTCh-RTCuZn sample. The numbers in sample labels corresponds to metallic zinc concentration in samples containing zinc. In samples containing copper, the numbers in sample labels indicate the metallic copper concentration and in HTChitosan, the numbers correspond to chitosan concentration. (The copper and chitosan concentrations were chosen to match those concentrations present in the zinc containing samples). Autoclaved water was used as control for killing cells and labeled as negative control. The growth of the cells in all the samples was normalized for the growth control wells and the viability was expressed as percentage growth compared to growth control wells. [* indicates significant difference between the treatment and growth control groups (*P* < 0.05)].

The murine macrophage cell line RAW 264.7 (immune cell) and the murine thymic epithelial cell line TE-71 (structural cell) were used to screen for any cytotoxic effect caused by chitosan–metal hydrogel. In case of RAW264.7 cells, only about 5% cells treated with HT chitosan–metal hydrogel were killed, whereas cells treated with just the mixture of copper and zinc salts (with no chitosan) exhibited significant reduction in viable cell numbers (92% of cells killed) at a metallic zinc concentration of 50 *μ*g/ml (Figure 5A). Though at lower metallic concentration (≤25 *μ*g/ml), the cytotoxicity levels were not significantly different between the samples. In TE-71 epithelial cells, the metals were more toxic and started killing cells at metallic zinc concentrations as low as 25 *μ*g/ml, whereas at the same concentration (25 *μ*g/ml), the chitosan-containing samples were not toxic to the cells (Figure 5B). The HT-chitosan matrix appears to be protective against metal derived cytotoxicity in two different cell lines (structural and immune cells) at least until the concentration of 25 *μ*g/ml of metallic zinc.

## Discussion

Commercially feasible potential alternatives are in greater need to reduce the total amount of classical antibiotics that are used as AGPs in food animal production facilities (19, 20). Metals, such as copper and zinc, are already used as salt feed supplements in cattle, swine, and chicken farms owing to their antimicrobial and growth promoting properties (10, 11, 21). In this report, we showed that a hydrothermally treated chitosan hydrogel loaded with copper and zinc metal particles have a growth inhibitory effect on both the “fermenters” group of gastric flora and the “opportunistic pathogen” group; target microbes of the antibiotics that are used as growth promoting feed supplements in animal farms (6). This study also showed that by using chitosan hydrogel matrix, any potential cytotoxic effect of the metals salts on the host tissue can be minimized.

In our previously published studies, we showed that HT treatment generates water-dispersible chitosan, a property that would allow for uniform mixing of chitosan with the animal feed in industrial scale livestock farms (18). The other advantage of HT treatment is that it increased the availability of functional groups that in turn allowed for embedding high amounts of copper (18) and zinc in a chitosan hydrogel matrix (Figures 1A–E). Also, we showed earlier that one of the advantages of hydrothermally treated chitosan was that it could alter the oxidation states of metals that were bound in them (18). Hydrogel formation after addition of metal salts to chitosan would allow for formation of a coating on the intestinal tract when fed as a feed additive that in turn would facilitate a larger surface area of metal nanoparticles interacting with gut microbiome. Moreover, the presence of metal nanoparticles in multiple oxidation states and forms (Figure 2) would provide the advantage of having multiple modes of killing gut microbes (22, 23); it would significantly reduce the possibility of development of resistance among microbes to these metals. This property in particular is superior over the current method of feeding copper sulfate and zinc oxide salts as they have been indicated in microbial resistance development (10). Multiple oxidation states of metals were not shown in earlier studies that used chitosan as either an encapsulating nanoparticle (14) or as a chelating material (17), suggesting the advantage of HT treatment over other methodologies for delivering transition metals for eliciting antimicrobial effect.

One of the functions of AGPs is to minimize the load of fermenting bacteria in the gut of the food animals such that minimal or no glucose and/or energy is lost via fermentation that can be converted into muscle weight otherwise. Particularly, *Lactobacillus* species of bacteria have been indicated as one of the primary targets of AGPs because they produce bile salt hydrolase, which has shown to have a negative impact on host animal digestion and absorption of nutrients (5). In this study, we used the strain, *L. fermentum* as a representative organism of *Lactobacillus* species to screen for antimicrobial effect of our chitosan–metal hydrogel (Figures 3 and 4). The presence of chitosan enhanced the antimicrobial effect of the metal salts significantly on *L. fermentum* as HTCh-RTCuZn completely inhibited the growth at zinc concentrations of 300 *μ*g/ml while RTCuZn (salts alone without chitosan) did not significantly inhibit the bacterial growth even at the same metallic zinc concentrations. The antimicrobial part of this study used both a metabolic activity based assay (alamar blue assay) and viability count (CFU) assay to cross-verify the effect on the microbes, since metabolic activity may not always correlate with the total number of viable bacteria. For example, in case of *E. coli* treated with HTCh-RTCuZn 800 sample, the dye reduction was statistically significant (Figure 3) but, when the viability of the bacteria was counted on a CFU assay (Figure 4), there was not a significant reduction in the viable bacterial count in the treated sample when compared to the growth control.

Another function of AGPs is to reduce the load of opportunistic and zoonotic pathogens group in the gut microbiome so that the animal’s immune system spends less energy on fighting these microbes. In this study, the chitosan–metal hydrogel reduced the representative model opportunistic pathogen load at comparable levels to the salt mixture suggesting minimal interference from chitosan on the antimicrobial effect of the metals (Figures 3 and 4). The advantage of optimal reduction in bacterial load and not a complete elimination of *E. coli*, *S. aureus*, and *E. faecalis* was that it would not significantly alter the balance between commensal beneficial gut microbes. The level of copper in these experiments is lower (57.6 *μ*g/ml) than that of our previous findings (18) where it required about 100–150 *μ*g/ml of metallic copper to exhibit a bacterial killing effect when copper was used alone with chitosan (18). A combination of copper and zinc provided an additive effect where only half the amount of either metal is required to elicit a similar antimicrobial effect that of the case in which the metals were used alone. Also, previous publications have shown that chitosan by themselves as microspheres were able to reduce *E. coli* shedding in feces of cattle (15); so we can speculate that the presence of chitosan has added to the cumulative antimicrobial effect. Additionally, Gram-negative organisms (*E. coli* and *E. faecalis*) were killed at higher concentrations (800 *μ*g/ml of metallic zinc) while Gram-positive organisms were killed at lower concentrations (400 *μ*g/ml of metallic zinc) suggesting a cell wall targeting activity of our chitosan–metal hydrogel (Figures 3 and 4) though we do not know the exact mechanism of killing yet. Though the concentrations considered were able to provide enhanced antimicrobial effect under *in vitro* conditions in a 96-well plate, further studies in target animals only would unveil the actual concentrations that would be good enough to elicit a growth promoting effect. Additionally, the difference between monogastric animals and ruminants in terms of the diversity of their gut microbial communities would also influence the effective concentration levels in target animals.

The HTCh-RTCuZn significantly reduced the cytotoxic effects of the metallic salts on two different types of eukaryotic cells (Figure 5). In both a phagocytic cell line (RAW264.7 macrophages) and in a structural cell line (TE-71 thymic epithelial cells), presence of chitosan appears to have provided a protective effect against toxicity of metals (Figure 5). The rationale behind the selected concentrations in cytotoxicity experiments (different from antibacterial assays) was to more closely resemble the (ratio of epithelial cell numbers to metallic concentration) actual animal gut microenvironment. When the same concentrations (100–800 *μ*g/ml of metallic zinc) considered in antimicrobial studies were used, all the samples (with and without chitosan) exhibited significant cytotoxicity in a 96-well plate (data not shown). The cytotoxicity exhibited at those concentrations was understandable considering the low number of macrophages/epithelial cells that were exposed per microgram of metal in a smaller surface area/volume. HT-chitosan alone did not exhibit significant cytotoxicity (at least until 28.8 *μ*g/ml). The toxicity to cell lines was primarily caused by the zinc concentration as the material containing HTCh-RTCu (HT chitosan embedded with copper alone) exhibited reduced toxicity (concentrations adjusted to reflect the copper concentration in the corresponding HTCh-RTCuZn samples) when compared to cells treated with HTCh-RTZn (at 50 *μ*g/ml) (Figure 5). Therefore, we can deduce that at higher metallic concentrations (used for growth promoting effect), embedding the copper and zinc in a HT treated chitosan matrix would reduce cytotoxicity to host animal cells. Since the HTCh-RTCuZn material exhibits significantly reduced cytotoxicity, we can speculate that when used as an antimicrobial feed additive in animals, it will elicit minimal or no inflammatory reaction that will in turn help in higher feed conversion ratio. Follow-up publication of this project will include optimization of synthesis protocol by altering the chitosan hydrolyzing components in preparing this chitosan–metal hydrogel and subsequent assessment of cytokine and other immunological responses in relevant eukaryotic cell lines. Further studies involving target animal models will include NRC recommended levels of minerals to accurately quantify the growth promoting effect of chitosan hydrogel-aided delivery of metals.

## Author Contributions

PR conceived and designed the study, collected data, performed critical analysis and interpretation of data, and wrote the manuscript. Also accepts to be accountable for all aspects of this work. SS helped in conception and design of the study, critical analysis, interpretation of data, and editing the manuscript.

## Conflict of Interest Statement

The authors declare that the research was conducted in the absence of any commercial or financial relationships that could be construed as a potential conflict of interest.
